# Smartphone Apps for Cardiovascular and Mental Health Care: Digital Cross-Sectional Analysis

**DOI:** 10.2196/63642

**Published:** 2025-11-13

**Authors:** Manjot Singh, Julian Herpertz, Noy Alon, Sarah Perret, John Torous, Daniel Kramer

**Affiliations:** 1 Richard A. and Susan F. Smith Center for Outcomes Research, Beth Israel Deaconess Medical Center, 375 Longwood Ave, Boston, MA, 02215, United States, 1 6176678800; 2 University of Connecticut School of Medicine, UConn Health, Farmington, CT, United States; 3 Division of Digital Psychiatry, Department of Psychiatry, Beth Israel Deaconess Medical Center, Boston, MA, United States

**Keywords:** smartphones, cardiovascular disease, cross sectional, application, app, smartphone app, cardiovascular, cardio, mental health, effectiveness, patient-reported symptoms, mental disorder, symptoms, effective

## Abstract

**Background:**

The rapidly expanding digital health landscape offers innovative opportunities for improving health care delivery and patient outcomes; however, regulatory and clinical frameworks for evaluating their key features, effectiveness, and outcomes are lacking. Cardiovascular and mental health apps represent 2 prominent categories within this space. While mental health apps have been extensively studied, limited research exists on the quality and effectiveness of cardiovascular care apps. Despite their potential, both categories of apps face criticism for a lack of clinical evidence, insufficient privacy safeguards, and underuse of smartphone-specific features alluding to larger shortcomings in the field.

**Objective:**

This study extends the use of the MINDApps framework to compare the quality of cardiovascular and mental health apps framework to compare the quality of cardiovascular and mental health apps with regard to data security, data collection, and evidence-based support to identify strengths, limitations, and broader shortcomings across these domains in the digital health landscape.

**Methods:**

We conducted a systematic review of the Apple App Store and Google Play Store, querying for cardiovascular care apps. Apps were included if they were updated within the past 90 days, available in English, and did not require a health care provider’s referral. Cardiovascular care apps were matched to mental health apps by platform compatibility and cost. Apps were evaluated using the M-Health Index & Navigation Database (MIND; MINDApps), a comprehensive tool based on the American Psychiatric Association’s app evaluation model. The framework includes 105 objective questions across 6 categories of quality, including privacy, clinical foundation, and engagement. Statistical differences between the 2 groups were assessed using two-proportion Z-tests.

**Results:**

In total, 48 cardiovascular care apps and 48 matched mental health apps were analyzed. The majority of apps in both categories included a privacy policy; yet, the majority in both samples shared user data with third-party companies. Evidence for effectiveness was limited, with only 2 (4%) cardiovascular care apps and 5 (10%) mental health apps meeting this criterion. Cardiovascular care apps were significantly more likely to be used in external devices such as smartphone-based electrocardiograms and blood pressure monitors.

**Conclusions:**

Both categories lack robust clinical foundations and face substantial privacy challenges. Cardiovascular apps have the potential to revolutionize patient monitoring; yet, their limited evidence base and privacy concerns highlight opportunities for improvement. Findings demonstrate the broader applicability of the MINDApps framework in evaluating apps across medical fields and stress the significant shortcomings in the app marketplace for cardiovascular and mental health. Future work should prioritize evidence-based app development, privacy safeguards, and the integration of innovative smartphone functionalities to ensure that health apps are safe and effective for patient use.

## Introduction

The rapidly growing digital health landscape enhances accessibility and broadens the dissemination of health-related products that were once limited to traditional medical settings [1]. According to recent reports by Quintiles and IMS Health Incorporated, more than 350,000 digital health apps are currently available on the Apple App Store and Google Play Store [2]. These include apps that connect to external devices, record and transmit patient health data, or use diagnostic sensors integrated into smartphones. With the growing number of apps on the market and an increasing number of users downloading them, there is an urgent need to evaluate their quality based on criteria such as evidence base, usability, data privacy, and security [3].

The US Food and Drug Administration (FDA) plays a key role in regulating the marketing of medical products and monitoring of safety signals. However, policies to ensure a reasonable assurance of safety and effectiveness’ for digital health apps remain underdeveloped [4]. Many apps marketed as tools for health improvement are categorized as health and wellness products, which exempts them from FDA regulation [5]. To address these gaps, the FDA recently concluded its precertification pilot program, aimed at streamlining the evaluation of new apps within resource constraints. Despite its innovative approach, the program yielded mixed results, hindered by the lack of formal legislative authority to enforce and implement new evaluation criteria [6]. Recognizing the need for standardized quality assessment, numerous frameworks for evaluating health apps have been proposed. Defined as criteria-based tools for assessing the quality of mobile health apps, a recent review identified 44 distinct frameworks, each varying in evaluation methodologies and quality criteria [7].

One of the most widely used frameworks is the American Psychiatric Association (APA) app evaluation model, which uses a hierarchical structure to assess apps based on key quality criteria: access and background, privacy and safety, clinical foundation, usability, and data integration toward therapeutic goals [8]. To operationalize this model, our team developed the M-Health Index & Navigation Database (MIND) platform—a user-friendly database that provides more than 100 yes-or-no answers to critical evaluation questions [9]. These include whether an app costs money to download, has a privacy policy, is supported by studies, offers real-time responses to enhance engagement, or integrates with electronic health records [10].

MINDApps has already been used in numerous studies evaluating the quality of mental health apps, establishing its quality standards as an excellent control group for comparison [10-12]. The quality of cardiovascular care apps has yet to be evaluated by MINDApps. Mobile health apps offer 2 key potential benefits in cardiology: they empower and engage individuals to make healthier lifestyle changes, and they enhance patient monitoring through features like electrocardiography and blood pressure tracking via wristbands and watches connected to an app [13]. Wearable devices integrated with apps are widely regarded as the future of cardiology, marking a clear shift toward more digitized and patient-centered care in the field [14-16]. With an increasing number of people relying on these apps and their functionality, it is crucial to evaluate their effectiveness, safety, and privacy standards. This is particularly important given recent literature indicating that the evidence base for these apps and the integration of wearable data into clinical cardiology are still in their early stages [14].

We conducted a systematic review of the Google Play Store and Apple App Store to identify smartphone apps used in cardiovascular care. Using the MINDApps framework, we evaluated these apps to assess their clinical foundation, privacy standards, security measures, platform compatibility, and cost. The results were then compared to the quality criteria of a comparable sample of mental health apps. We hypothesized that only a minority of both cardiovascular care and mental health apps would be supported by evidence, that the majority of apps would share personal health data with third-party companies, and that cardiovascular care apps would be used in external devices more frequently than mental health apps. We aim to identify and compare the limitations in both mental health and cardiovascular care apps by extending the application of the MINDApps framework to highlight areas within the broader digital health landscape that require improvement.

## Methods

### App Identification and Selection Criteria

The Apple App Store and Google Play Store were queried the for cardiovascular apps from January 19, 2023, to March 16, 2023, using the search terms “Blood pressure,” “Heart,” and “Cardiovascular,” yielding a total of 159 apps across both sites. Terms were selected to mimic patient and provider search terms when identifying a potential assistive app. Apps were eligible for inclusion if they were updated within the past 90 days and in English. We excluded apps that required referral from a health care provider, had not been updated in the past 90 days, or were otherwise not functional. These limits were applied to ensure user accessibility, app functionality, and recency. Information was collected via app home pages and by trialing each app.

Cardiology apps were then rated using the MINDApps framework. Cardiology apps were matched to a subset of mental health apps from the MIND database by considering platform compatibility, such as whether the apps were available only on Android, only on iOS, on both Android and iOS, or across Android, iOS, and web platforms. To closely mimic the functionality and intention of cardiology apps, the mental health apps were chosen from the MINDApps database, which included journaling/mood trackers as they reflect the functionality of cardiology apps better than apps designed to provide a psychotherapy intervention. Additionally, apps were matched based on their cost structure (totally free vs some form of payment) and platform available (Apple App Store, Google Play Store, or both). No citation chaining, registry searching, or developer contacting was conducted, as this study focused solely on publicly available app marketplace content.

### Evaluation Framework and Statistical Analysis

MINDApps is the largest publicly available database of mental health apps [17]. The index consists of 105 objective questions (yes/no) based on the APA’s app evaluation model and one free-text section for reviewers to add any additional information [9]. Apps are evaluated across six categories: (1) app origin and accessibility, (2) privacy and security, (3) clinical foundation, (4) features and engagement, (5) inputs and outputs, and (6) interoperability (see Multimedia Appendix 1 for the full list of questions). Reviews are conducted by evaluators who have undergone interrater reliability training. App ratings are updated every 6 months recognizing the fast dynamic of the health app marketplace. Using the MINDApps framework, reviewers having undergone the same interrater reliability training evaluated the identified cardiovascular care apps by applying its 105 questions, which assess key app features such as privacy policies, clinical foundation, and engagement. For example, one question asks whether the app includes a privacy policy, allowing us to determine the proportion of cardiovascular care apps that meet this basic privacy standard. We then compared the results with those of mental health apps to identify differences in quality criteria. Statistical differences between the 2 groups were assessed using two-proportion Z-tests.

## Results

### App Overview

**Figure 1.**
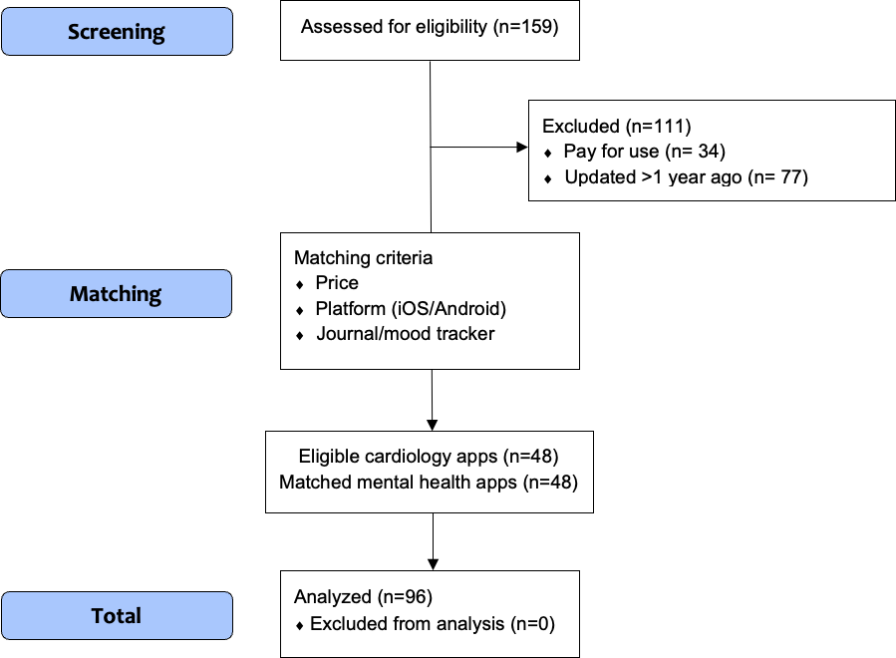
App selection Process.

We identified 48 cardiovascular care apps on the Apple App store and Google Play store, which met the eligibility criteria (Table 1). These apps were compared to 48 mental health apps derived from MINDApps. Refer to Multimedia Appendix 2 for the full list of included apps. The app selection process is shown in Figure 1.

**Table 1.** Comparing the quality criteria of cardiovascular care and mental health apps.

Category	Cardiology apps, n (%)	Mental health apps, n (%)	*P* value
Total number of apps	48 (100)	48 (100)	—^a^
Data security			
No privacy policy	6 (12.50)	5 (10.42)	>.99
Sharing PHI^b^ with third party	30 (62.50)	28 (58.33)	.68
Cost			
Completely free option	12 (25.00)	11 (22.92)	.81
For profit	48 (100)	44 (91.67)	.04^c^
Platform			
Android	13 (27.08)	8 (16.67)	.22
iOS	22 (45.83)	17 (35.42)	.30
Android + iOS	13 (27.08)	21 (43.75)	.09
Data collection			
Survey/diary	39 (81.25)	46 (95.83)	.03^c^
Microphone	2 (4.17)	18 (37.50)	<.001^d^
Step count	5 (10.42)	3 (6.25)	.46
Camera	21 (43.75)	19 (39.58)	.68
Requires external device	14 (29.17)	6 (12.50)	.04^c^
Reports summary of data	44 (91.67)	44 (91.67)	>.99
Email or export data	39 (81.25)	32 (66.67)	.10
Clinical foundation	2 (4.17)	5 (10.42)	.24
Features			
Biodata	30 (62.50)	0 (0)	<.001^d^
Biodata with feedback	12 (25.00)	1 (2.08)	.001^e^
Symptom tracking	34 (70.83)	47 (97.92)	<.001^d^

a Not applicable.

b PHI: patient health information.

c *P*<.05.

d *P*<.001.

e *P*<.01.

### Data Security and Privacy

**Figure 2.**
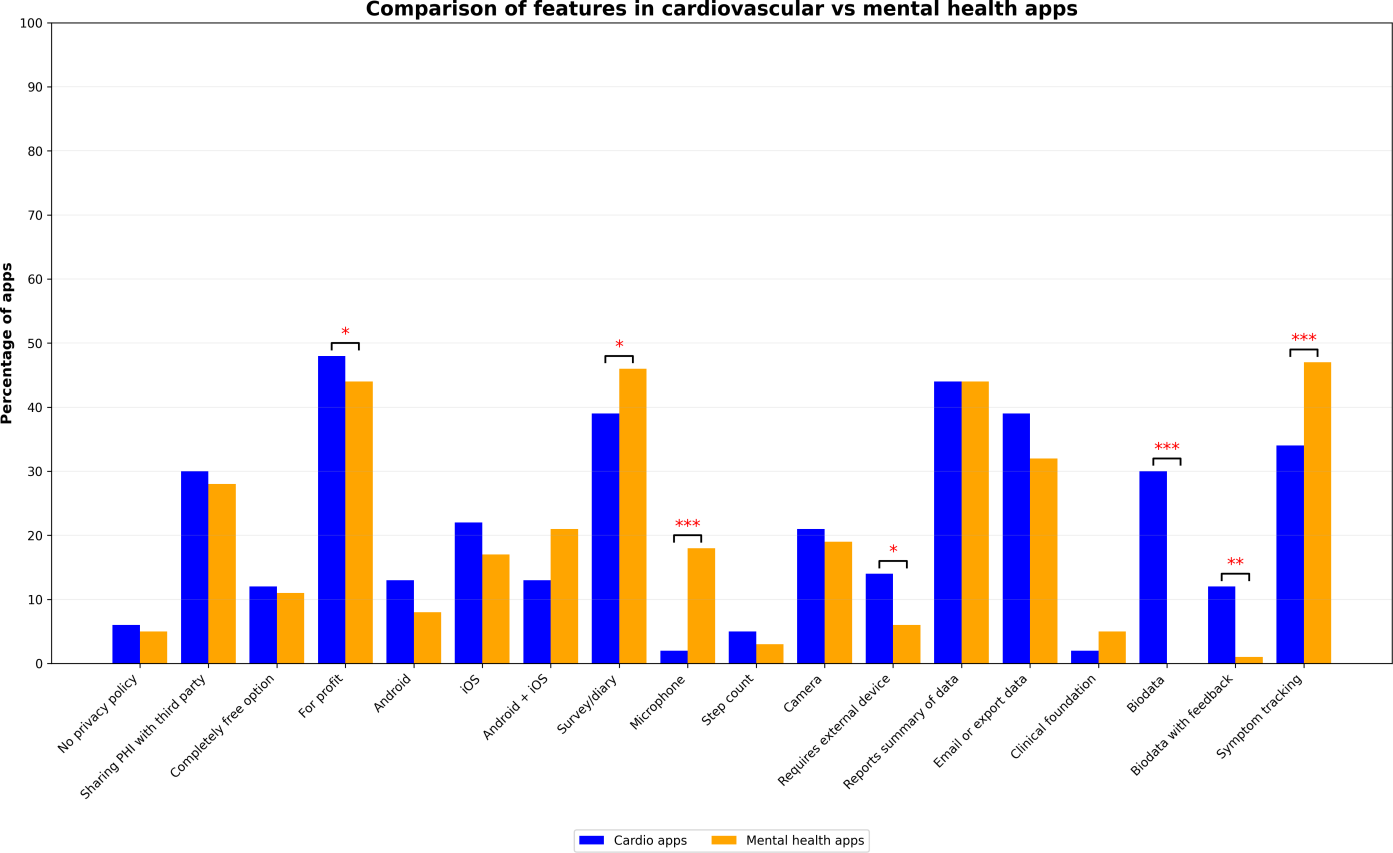
Comparison of features in cardiovascular vs mental health apps. PHI: patient health information. *P<.05, **P<.01, ***P<.001.

The majority of apps in both the cardiovascular care and mental health samples had a privacy policy. However, many of these apps disclosed user data to third-party companies (Table 1). A significantly higher proportion of cardiovascular care apps was developed by for-profit companies compared to the mental health app sample (*P*=.04; Figure 2): 44 of all 48 (92%) mental health apps were developed by a for-profit company, while all of the 48 identified cardiovascular care apps originated from a for-profit company.

### Features and Functionality

Apps in both samples used similar features to assess user data. However, it was significantly more likely for mental health apps to collect speech data using the smartphone’s microphone than it was for the cardiovascular care apps (*P*<.001). Cardiovascular care apps on the other hand had a higher likelihood of being connected to an external device (*P*=.04). In terms of features, a significantly higher proportion of cardiovascular care apps used biodata (*P*<.001), whereas mental health apps were significantly more likely to include symptom tracking capabilities (*P*<.001).

### Clinical Foundation and Evidence

A minority of apps in both samples provided evidence for their effectiveness, with only 2 of all 48 (4%) cardiovascular care apps and 5 of all 48 (10%) mental health apps meeting this criterion.

## Discussion

### Summary of Key Findings

Both cardiovascular and mental health apps suffer from limited clinical validation and substantial data sharing practices. While cardiovascular apps excel in integration with external devices and bio capture, they lack evidence in support and transparency. Both classes of apps lack supporting clinical evidence while including a large price tag, highlighting the importance of need for regulation and counseling for integrated patient care use.

### Limitations in Technology Integration and Data Privacy in Health Apps

We found that the current apps targeted to cardiovascular conditions and mental health both fail to leverage new technology for data collection. The majority of cardiology and mental health apps collect data through diaries and surveys, which rely on self-report and/or users being able to obtain their vitals independently from the app. Though monitoring and tracking symptoms in real time can be beneficial to patients, these “digital notebooks” do not robustly use functionalities that are unique to smartphones (ie, microphones, cameras, and sensors) [18-20]. In behavioral health, research teams continue to innovate how the sensors in smartphones can be used to monitor patients’ symptoms, behaviors, and environment in real time; yet, there is little translation from the academic literature to marketplace options [21-23]. Integrating the data streams and functionalities that are unique to smartphones can help marketplace options present more innovative interventions for health conditions [18,21-23].

A major concern with health apps, which is reflected in our hypotheses, is the sharing of patient health information with third-party companies, which would typically be protected in standard health care settings [24-26]. Our findings highlight this issue, with the majority of both cardiovascular and mental health apps sharing data with third-party companies. Recent investigations on mental health apps reported similar results [10]. In our study, we demonstrate that this issue is not exclusive to the field of mental health, as cardiovascular care apps also face significant challenges in ensuring data ownership. The lack of evidence for supporting effectiveness is another frequently reported issue [27,28]. With only about one-tenth of mental health apps and fewer than one-twentieth of cardiovascular care apps in our sample providing evidence for their effectiveness, it becomes clear why the health app marketplace is often likened to a “wild west” of medical software [29,30]. Cardiovascular care apps are more likely to integrate with external devices than are mental health apps, which is unsurprising given that many are equipped with smartphone-based electrocardiograms or blood pressure monitoring functions [31,32]. These external devices have the potential to revolutionize patient monitoring in cardiology. However, without app developers providing evidence of their feasibility and efficacy, we remain cautious about the current status of health app adoption in this field.

### Future Directions and Quality Assurance in Health Apps

According to data from the Medical Expenditure Panel Survey, heart disease in the United States incurred an estimated cost of $229 billion between 2017 and 2018, encompassing health care services, medications, and lost productivity [33]. Digital health apps may offer opportunities to mitigate this public health challenge by providing more streamlined access to care. The current app ecosystem, however, exhibits significant shortcomings. Despite the concerns, we are convinced that health apps will continue to grow in prominence in society and health care. As medicine becomes increasingly digitized, ensuring its quality assurance is indispensable. App libraries such as MINDApps are able to alleviate concerns and increase trust in health apps. Patients have expressed a desire for app libraries to provide personalized recommendations tailored to their needs [34]. MINDApps already provides personalized recommendations in the field of mental health, and in this study, we demonstrate that its framework can be effectively applied to other medical fields. We present the first use case of MINDApps being adapted to cardiovascular health apps, demonstrating that this framework can be effectively extended beyond mental health to categorize available apps and assist patients and consumers in making informed decisions when selecting health apps.

### Limitations

This study has several limitations: we included only apps that provided content in English, which may limit generalizability. Additionally, by including only apps updated within the last 90 days, there is a potential for selection bias and, thus, this sample may not represent the full range of available apps, potentially excluding newer or less popular options that offer better or different features. Furthermore, as a cross-sectional study, the included apps may have been updated or modified since the time of analysis and publication. While this study provides insights into the current state of app quality and features, it cannot determine causality or the long-term effectiveness of whether the use of these apps leads to sustained improvements in health outcomes over time.

### Conclusion

Our findings reveal that the current app marketplace for cardiovascular and mental health on both the Apple App Store and Google Play Store is built on a limited clinical foundation and presents significant privacy and security concerns. We argue that there is need for better-quality assurance of health apps. MINDApps provides valuable guidance in identifying effective and secure apps tailored to the individual preferences of both clinicians and users. In this study, we demonstrate that this capability extends beyond the field of mental health, showing that the framework can be successfully applied to other medical domains as well.

## Supplementary material

10.2196/63642 Multimedia Appendix 1 List of questions.

10.2196/63642 Multimedia Appendix 2 List of included apps.

## Abbreviations

APA: American Psychiatric Association
FDA: Food and Drug Administration
MIND: M-Health Index & Navigation Database
