# Supplementary material for: Smartphone Apps for Cardiovascular and Mental Health Care: Digital Cross-Sectional Analysis
Source: JMIR Mhealth Uhealth. 2025 Nov 13;13:e63642. doi: 10.2196/63642 (PMC12614660; doi:10.2196/63642)
Supplement: Multimedia Appendix 1 [file mhealth-v13-e63642-s001.docx]

**Smartphone Apps for Cardiovascular Care Compared with those for Mental Health:**

**A Cross-Sectional Analysis**

**Appendix 1 Questions**

**Platforms**

1. Android
2. iOS
3. Web

**Supported Conditions**

1. Indicate the app's supported conditions

**Cost**

1. Is the app free to download?
2. Is the app totally free?
3. Is there a one-time payment?
4. Are there in-app purchases?
5. Is it a subscription (recurrent/monthly/annual)?

**App Origin**

1. Does it come from the government?
2. Does it come from a for-profit company or developer?
3. Does it come from a non-profit company?
4. Does it come from a trusted healthcare company?
5. Does it come from an academic institution?

**Privacy & Security**

1. Is there a privacy policy?
2. Is the user data stored only on the device?
3. Is the user data stored on a server?
4. Can you delete your data?
5. Does the app declare data use and purpose?
6. Does the app report security measures in place?
7. Is PHI (Protected Health Information) shared?
8. Is de-identified/anonymized data shared?
9. Is aggregate data shared?
10. Can you opt out of data collection?
11. Does the app claim it meets HIPAA?
12. Reading level of the privacy policy (what grade reading level)?

**Functionality and Data Sharing**

1. Does it work with Spanish?
2. Does the app work offline?
3. Does it have at least one accessibility feature (e.g., adjust text size, text to voice, colorblind-friendly color scheme)?
4. Do you own your data?
5. Can you email or export your data?
6. Can you send your data to a medical record?

**Evidence & Clinical Foundation**

1. Is the app content well-written, correct, and relevant?
2. Does the app appear to do what it claims to do?
3. Is the app patient-facing?
4. Can the app cause harm?
5. Does the app specify that it is not a replacement for medical care?
6. In the case of an emergency, does the app appropriately advise the patient?
7. Does the app contain supporting studies?
   1. How many feasibility/usability studies?
   2. What is the highest feasibility impact factor?
   3. How many evidence/efficacy studies?
   4. What is the highest efficacy impact factor?

**Features**

1. Mood tracking?
2. Medication tracking?
3. Sleep tracking?
4. Symptom tracking?
5. Productivity?
6. Physical exercise tracking?
7. Psychoeducation?
8. Journaling?
9. Mindfulness?
10. Deep breathing?
11. Picture gallery/hope board?
12. iCBT or sleep therapy?
13. CBT?
14. ACT?
15. DBT?
16. Peer support?
17. Connection to coach/therapist?
18. Biodata?
19. Goal setting/habits?
20. Physical health exercises?
21. Chatbot interaction (with virtual character)?
22. Biofeedback with sense data (e.g., EEG, HRV, skin conductance)?
23. Identify new condition?

**Engagement Style**

1. User-generated data?
2. Chat/message-based?
3. Is it a screener/assessment?
4. Real-time response?
5. Asynchronous response?
6. Gamification (points, badges)?
7. Videos?
8. Audio/music/scripts?
9. AI support?
10. Peer support?
11. Network support?
12. Collaborative with provider/other?

**Inputs**

1. Surveys?
2. Diary?
3. Location?
4. Contact list?
5. Camera?
6. Microphone?
7. Step count?
8. External devices (e.g., wearable sending direct data)?
9. Social network?

**Outputs**

1. Notifications?
2. References/information?
3. Social network?
4. Reminders?
5. Graphs of data?
6. Summary of data (text or numbers)?
7. Link to formal care/coaching?

**App Use**

1. Is it a self-help/self-management tool?
2. Is it a reference app?
3. Is it intended for hybrid use with a clinician in conjunction with a treatment plan?
