# Supplementary material for: Smartphone Apps for Cardiovascular and Mental Health Care: Digital Cross-Sectional Analysis
Source: JMIR Mhealth Uhealth. 2025 Nov 13;13:e63642. doi: 10.2196/63642 (PMC12614660; doi:10.2196/63642)
Supplement: Multimedia Appendix 2 [file mhealth-v13-e63642-s002.docx]

**Appendix 1, Applications**

Cardiology Applications

1. Blood Pressure Tracker
2. SmartBP Blood Pressure
3. Heart Analyzer
4. Instant Heart Rate: HR Monitor
5. Pulse Plis: Heart Rate
6. Tonome: Blood Pressure Monitor
7. Cardiograph Heart Rate
8. Photo Afib Detector (Lite)
9. Blood Pressure Log – MyDiary
10. Heart Rate ECG Pulse
11. Blood Pressure Pro+
12. Blood Pressure AppPro
13. Blood Pressure Calculator
14. Hands Free Heart Rate Monitor
15. Heart Scan: Heart Rate Monitor
16. Afib 2gether
17. Blood Pressure Monitor
18. Blood Pressure Diary
19. Blood Pressure - Cardio Journal
20. Blood Pressure (BP) Watch
21. Blood Pressure App: High & Low
22. Blood Pressure
23. Heart Rate Plus: Pulse Monitor
24. QALY
25. Qardio
26. Heart Rate Monitor
27. Kardia
28. AVAX Blood
29. HRV: Heart Rate Cardio Monitor
30. Blood Pressure Tracker+
31. Equate
32. InPulse
33. Cardio: Heart Rate Monitor
34. AFibCheck
35. HealthRate: AI Pulse Monitor
36. Heartify
37. Blood Pressure App++
38. Sanket Life
39. Heart Rate Monitor: Pulse
40. HRV Camera ECG BLE
41. Heart Rate Monitor
42. Heart Rate PRO - Healthy Pulse
43. Blood Pressure Monitor: Bp App
44. Heart rate Monitor: Pulse Monitor +
45. Blood Pressure Monitor – iCare
46. Blood Pressure App & Log
47. Welltory
48. Spandan-ECG/EKG on Smartphone

Mental Health Applications

1. Mood Log
2. Daylio Journal - Mood Tracker
3. Moodwell: Moody Tracker Diary
4. Mooditude — A Happier You!
5. Moody: Mood Tracker and Journal
6. Moodnotes - Mood Tracker
7. Moods: Mental Health Tracking
8. Easy Mood Diary
9. Mood Tracker Journal
10. Mood Care Journal - Self Tracker
11. Mood Balance Self Care Tracker
12. UP! - Depression, Bipolar & Bo
13. Aetheria
14. Mood Ring: Your Emoji Journal
15. Anxiety Tracker-Log & Analyze
16. EmojiTrack+: Monitor Moods, Me
17. Reflectly: Mood Tracker Diary
18. Moodily - Mood Tracker, Depression
19. eMoods Bipolar Mood Tracker
20. MoodTools - Depression Aid
21. DailyBean: Simplest Journal
22. Panic Diary: An Anxiety Tracker
23. Remente: Self Care, Wellbeing
24. uMore - Mental Health Tracker
25. Moodflow: Mood Tracker
26. Symptom & Mood Tracker
27. Breeze: Mental Health
28. Sanvello: Anxiety or Depression
29. Moodicious Lite: Your All in One Mood Tracker, Mood Diary, and Mood Analyzer
30. Notes Motivation Mood Journal
31. Don't Panic
32. Pond Self Care Journal
33. #SelfLove (GG Confidence & Self-Love)
34. Worry Watch
35. Flamingo - Mood Tracker
36. Uplift
37. Tochi - Mood Tracker, Journal
38. Self-Help App for the Mind SAM
39. Personal Zen
40. MoodPanda Mood Diary
41. The Zone: Daily Self Care & Gratitude
42. Bipolar Mood Tracker
43. Bipolar UK Mood Tracker
44. MyPossibleSelf: Mental Health
45. Gratitude Self Care Journal
46. Lift: Anxiety or Depression
47. Anxiety Relief by Mindease
48. ‎MyPossibleSelf: Mental Health
